# Influenza Virus with Increased pH of Hemagglutinin Activation Has Improved Replication in Cell Culture but at the Cost of Infectivity in Human Airway Epithelium

**DOI:** 10.1128/JVI.00058-19

**Published:** 2019-08-13

**Authors:** Anika Singanayagam, Maria Zambon, Wendy S. Barclay

**Affiliations:** a Department of Medicine, Imperial College, London, United Kingdom; b Public Health England, Colindale, United Kingdom; Icahn School of Medicine at Mount Sinai

**Keywords:** influenza, hemagglutinin, viral replication, virology

## Abstract

Pandemic H1N1 (pH1N1) influenza virus emerged from swine in 2009 with an adequate capability to infect and transmit between people. In subsequent years, it has circulated as a seasonal virus and evolved further human-adapting mutations. Mutations in the hemagglutinin (HA) stalk that increase pH stability have been associated with human adaptation and airborne transmission of pH1N1 virus. Yet, our understanding of how pH stability impacts virus-host interactions is incomplete. Here, using recombinant viruses with point mutations that alter the pH stability of pH1N1 HA, we found distinct effects on virus phenotypes in different experimental models. Increased pH sensitivity enabled viruses to uncoat in endosomes more efficiently, manifesting as increased replication rate in typical continuous cell cultures under single-cycle conditions. A more acid-labile HA also conferred a small reduction in sensitivity to antiviral therapeutics that act at the pH-sensitive HA fusion step. Conversely, in primary human airway epithelium cultured at the air-liquid interface, increased pH sensitivity attenuated multicycle viral replication by compromising virus survival in the extracellular microenvironment. In a mouse model of influenza pathogenicity, there was an optimum HA activation pH, and viruses with either more- or less-pH-stable HA were less virulent. Opposing pressures inside and outside the host cell that determine pH stability may influence zoonotic potential. The distinct effects that changes in pH stability exert on viral phenotypes underscore the importance of using the most appropriate systems for assessing virus titer and fitness, which has implications for vaccine manufacture, antiviral drug development, and pandemic risk assessment.

**IMPORTANCE** The pH stability of the hemagglutinin surface protein varies between different influenza strains and subtypes and can affect the virus’ ability to replicate and transmit. Here, we demonstrate a delicate balance that the virus strikes within and without the target cell. We show that a pH-stable hemagglutinin enables a human influenza virus to replicate more effectively in human airway cells and mouse lungs by facilitating virus survival in the extracellular environment of the upper respiratory tract. Conversely, after entering target cells, being more pH stable confers a relative disadvantage, resulting in less efficient delivery of the viral genome to the host cell nucleus. Since the balance we describe will be affected differently in different host environments, it may restrict a virus’ ability to cross species. In addition, our findings imply that different influenza viruses may show variation in how well they are controlled by antiviral strategies targeting pH-dependent steps in the virus replication cycle.

## Abstract (precis)

The pH stability of the hemagglutinin surface protein varies between different influenza strains and subtypes and can affect the virus’ ability to replicate and transmit. Here, we demonstrate a delicate balance that the virus strikes within and without the target cell. We show that a pH-stable hemagglutinin enables a human influenza virus to replicate more effectively in human airway cells and mouse lungs by facilitating virus survival in the extracellular environment of the upper respiratory tract. Conversely, after entering target cells, being more pH stable confers a relative disadvantage, resulting in less efficient delivery of the viral genome to the host cell nucleus. Since the balance we describe will be affected differently in different host environments, it may restrict a virus’ ability to cross species. In addition, our findings imply that different influenza viruses may show variation in how well they are controlled by antiviral strategies targeting pH-dependent steps in the virus replication cycle.

## INTRODUCTION

Influenza viruses infect 10 to 20% of the population annually, causing a wide spectrum of disease from asymptomatic to life-threatening illness (1). These viruses are highly diverse, a consequence of their high mutation rate, low fidelity replication, and ability to reassort. Influenza viruses exist in animal reservoirs and can jump between animal and human hosts. If a novel strain acquires the ability to efficiently infect and transmit between people, a pandemic can arise.

When a novel influenza virus enters human circulation from the animal reservoir, mutations in polymerase genes enable efficient replication in human cells (2, 3), and mutations in the hemagglutinin (HA) surface protein enable binding to the α2,6-linked sialic acid receptors prevalent in the human respiratory tract (4). More recently, research has identified that HA protein stability is strongly selected for during human adaptation, suggesting that a stable HA is an important property for an influenza virus to become a successful human pathogen (5–7). Additionally, mutations that stabilized HA were required for the airborne transmission of H5N1 and pandemic H1N1 (pH1N1) influenza viruses between ferrets (5, 8–10).

Influenza virus HA is a homotrimer that binds to sialic acid receptors on the host cell surface, initiating viral entry by endocytosis. The incoming virion is trafficked from early (∼pH 6) to late (∼pH 5) endosomes. A threshold of acidic pH triggers the HA protein to undergo an irreversible conformational change, exposing the fusion peptide. This critical process initiates virus-host fusion, uncoating, and release of the viral genome into the cytoplasm. Diversity exists in the pH threshold at which the HA protein is triggered to fuse between different influenza virus strains and subtypes. Highly pathogenic H5N1 and H7N9 strains have a higher pH of fusion (5.6 to 6.0), whereas seasonal human strains are more acid stable (pH of fusion 5.0 to 5.3) (7, 11). Interestingly, the 2009 pH1N1 virus had an intermediate fusion pH of 5.5 when it first emerged in humans.

The stalk region of HA is relatively conserved across influenza virus subtypes and assumed to be mutationally intolerant (12, 13), although this may be strain and subtype specific (14). This has led to the development of a variety of promising HA stalk-targeting anti-influenza therapeutics that bind to epitopes critical for HA fusion (15–17). Escape mutations to stalk-targeting antivirals have been described in some preclinical studies (18–25), although the clinical consequences and accompanying effects on virus phenotype are not fully understood. Mutations in regions of HA critical for fusion can either stabilize or destabilize its structure and alter the activation threshold (26). Such mutations have been shown to arise and fix during the natural evolution of influenza viruses, suggesting a positive effect on functionality (5, 6). However, our understanding of when and why HA pH stability is advantageous to virus and how changes in the pH of HA activation influence virus behavior is incomplete.

In this study, we systematically investigated the consequences of point mutations that alter pH stability of the pH1N1 2009 virus. We found that single amino acid changes that affect HA activation pH can have strikingly differing impacts on virus phenotypes in different settings. We demonstrate that the pH environment encountered both inside and outside the host cell affects the replicative fitness of viruses with different acid stability. Our results add to the understanding of the biological importance and consequences of HA pH stability, which can aid in predicting and risk assessing for pandemic viruses and have translational impact in regards to the use and development of antiviral drugs and vaccines for influenza.

## RESULTS

### Generation of influenza viruses with mutations in the hemagglutinin stalk region that alter the pH of HA activation.

**FIG 1:**
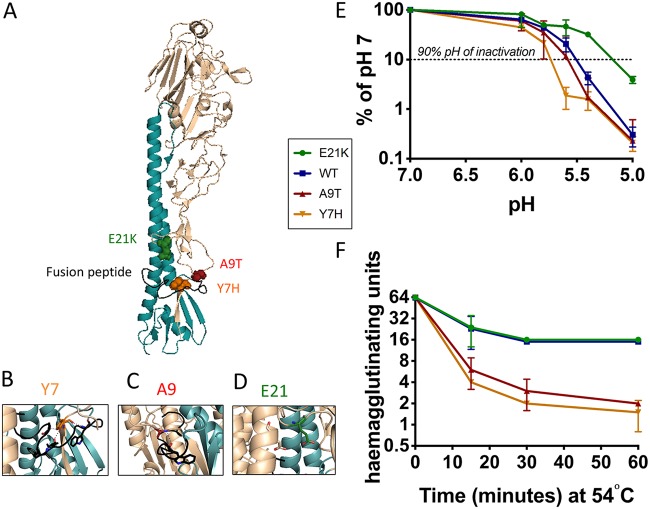
Generation of pH1N1 viruses with point mutations in the HA stalk that lead to altered acid and temperature stability. Three mutations predicted to alter HA pH stability were engineered into pH1N1 HA. (A) Residues in HA1 at positions 7, 9, and 21 (H1 numbering using mature HA sequence described by Burke and Smith [64]), located in close proximity to the fusion peptide (colored black), were selected. The location of mutations are modeled onto an HA monomer using the PyMOL molecular visualization tool (PDB ID 3LZG). (B) The hydroxyl group of tyrosine at position 7 in HA1 forms hydrogen bonds with the fusion peptide, which is predicted to destabilize HA when mutated to histidine (previously described in references 5 and 64). (C) The hydrophobic alanine residue at position 9 in HA1 interacts with W14 in the fusion peptide. Mutation to threonine is predicted to result in the destabilization of HA. A9T mutation was detected during cell culture passage experiments in our laboratory (A. Singanayagam and W. Barclay, unpublished data). (D) Residue 21 in HA1 is predicted to form a stabilizing intermonomer salt bridge with HA2 residue E47 when mutated from E to K (previously described in reference 42). (E) Virus was incubated in low-pH buffers for 15 min and the remaining infectious virus titrated by a plaque assay on MDCK cells. (F) Virus was incubated at 54°C for the indicated time and the remaining virus titrated by hemagglutination assay. (E and F) Data represent the mean ± standard deviation of triplicate experiments. Experiments are representative of at least two biological replicates.

We generated recombinant viruses from a typical first-wave 2009 pandemic H1N1 virus with point mutations in the HA stalk (Fig. 1A to D). Two mutations were previously reported in the literature (Table 1), and the third was detected in our laboratory during cell culture passage (our unpublished data). The viruses were identical in all seven other genes. Viral stocks had similar particle-to-PFU ratios (Table 1) and were passaged minimally after rescue. The mutant viruses were selected for study because they exhibited a wide range of HA activation pHs (Table 1). Consistent with previous studies (5, 11), the wild-type (WT) HA had a fusion pH of 5.5. The destabilizing Y7H and A9T mutations caused an increase in the pH of fusion to 5.9 and 5.8, respectively. The E21K mutation stabilized the HA to a pH of fusion of 5.3. We also tested the rate of viral inactivation in pH-adjusted buffers and calculated the 90% inhibitory concentration (IC_90_) pH of the HA mutants: 5.75 (Y7H mutant), 5.55 (A9T mutant), 5.45 (WT), and 5.15 (E21K mutant) (Fig. 1E and Table 1). In an experimental setting, elevated temperature is also capable of inducing conformational rearrangement of the HA molecule (27). We therefore tested the thermostability of each virus. After incubation at 54°C, the hemagglutinating activity of the Y7H and A9T mutant viruses was more rapidly lost than for the WT or the E21K mutant (Fig. 1F and Table 1). Taken together, our data confirm a hierarchy of viral stability determined by HA from least to most stable mutations of Y7H, A9T, WT, and E21K.

**TABLE 1:** Characteristics of HA mutant viruses^*a*^

Wild type or mutation	Particle/PFU ratio	pH of fusion (syncytial formation)	90% pH of inactivation	Thermostability (fold change at 30 min)	Source or reference
E21K	2.0	5.3	5.15	4	Cotter et al. (42)
Wild type	1.5	5.5	5.45	4	
A9T	1.8	5.8	5.55	21	Arose in cell culture passage (our unpublished data)
Y7H	3.5	5.9	5.75	32	Russier et al. (5), Zaraket et al. (51), Reed et al. (55), Thoennes et al. (65)

a Viral RNA content of viral stocks was calculated by a real-time quantitative PCR (RT-qPCR) and the PFU calculated by a plaque assay on MDCK cells. The stability of the viral mutants was assessed by syncytial formation, acid inactivation, and thermostability assays.

### HA stalk mutations that increase the HA activation pH enable virus to uncoat in endosomes more efficiently.

**FIG 2:**
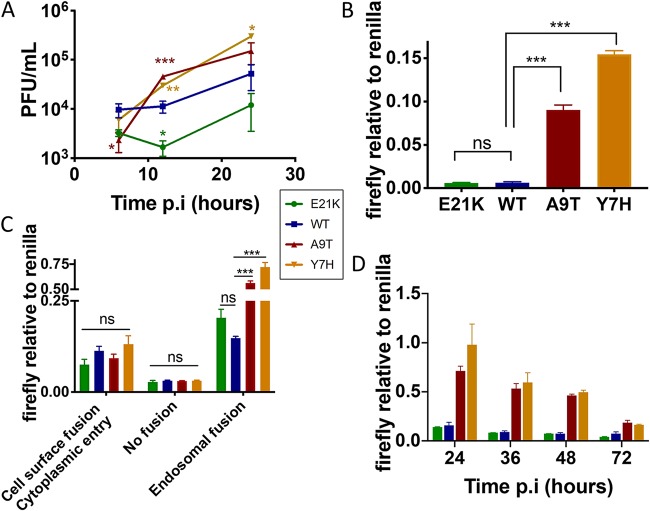
Early stages of viral replication. (A) A549 cells were infected with each HA mutant virus at high multiplicity of infection (MOI, 3). Infected cell supernatants collected at 6, 12, and 24 h postinfection (p.i) were titrated by plaque assay on MDCK cells. (B) A virus-driven replicon assay was performed in A549 cells infected with each virus at equal MOI, in triplicate. Luciferase signals were quantified at 24 h p.i and the data expressed as a ratio of firefly to *Renilla* luciferase levels. (C) Acid bypass assay was performed in A549 cells to compare virus-driven replicon expression following cytoplasmic fusion versus endosomal fusion. A negative no-fusion control was performed in conjunction. Luciferase signals were quantified at 24 h p.i., and the data are expressed as a ratio of firefly to *Renilla* luciferase levels. (D) A temporal analysis of virus-driven replicon reporter gene expression was carried out in 293T cells infected with each virus at equal MOI over 72 h. For all experiments, one-way ANOVA with Tukey’s posttest was used to compare wild-type (WT) virus to the other viruses. *, *P* < 0.05; **, *P* < 0.01; ***, *P* < 0.001; ns, not significant. Data represent the mean values from triplicate experiments, and error bars show the standard deviation. Experiments are representative of at least two biological replicates.

We infected immortalized human lung epithelial (A549) cells with the HA mutant viruses at a high multiplicity to allow for a single cycle of replication. The A9T and Y7H mutant viruses attained higher titers and at earlier time points than did the more-stable E21K and WT mutant viruses (Fig. 2A). The same pattern was seen in MDCK cells infected at high multiplicity (data not shown). In a virus-driven replicon assay in which a luciferase-encoding viral-like RNA was amplified and expressed by each virus infected at an equal MOI (as determined by plaque assay on MDCK cells), the Y7H and A9T mutants resulted in a significantly higher signal (Fig. 2B). This pattern was also evident when the assay was performed in 293T cells (data not shown). This suggests that the replicative advantage shown by the Y7H and A9T mutants was occurring at an early stage in the viral replication cycle. We could pinpoint this replicative advantage to virus uncoating in endosomes. The Y7H and A9T mutants, with high HA activation pH, stimulated increased production of firefly luciferase in the reporter assay only when viruses entered via fusion in endosomes and not when viruses were induced to fuse at the cell surface (Fig. 2C). This suggests that having a higher pH of HA activation enables influenza viruses to more efficiently uncoat in endosomes and release their genomes to the nucleus. When cells were incubated for longer, the WT and E21K mutant signal did not achieve the same levels as for the A9T and Y7H mutants, probably because a proportion of the virions entering the cell were lost to lysosomal degradation (Fig. 2D).

### In primary human airway epithelial cells, increased pH stability confers a replicative fitness advantage.

Strikingly, we found a very different fitness hierarchy when we infected primary human airway epithelial (pHAE) cells. pHAE cells, cultured at the air-liquid interface (ALI), are a fully differentiated primary cell model that mimics the morphological and physiological features of the human airway, including beating cilia, mucus production, and active ion transport. pHAEs are therefore a more relevant experimental model for influenza virus in the human upper respiratory tract than are traditional immortalized cell culture systems.

**FIG 3:**
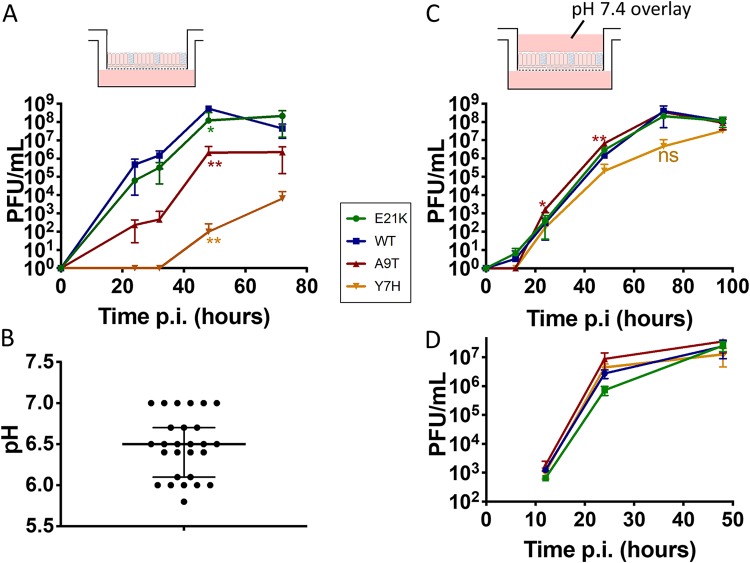
Replicative ability in primary human airway epithelial cells cultured at the air-liquid interface. Primary human airway epithelial (pHAE) cells (A and C) or MDCK cells (D) were infected with each virus at low MOI, in triplicate. Time points taken at intervals postinfection were titrated by plaque assay. (C) A liquid overlay buffered to pH 7.4 was maintained on the apical surface. One-way ANOVA with Tukey’s posttest was used to compare WT virus to the other viruses. *, *P* < 0.05, **, *P* < 0.01; ***, *P* < 0.001; ns, not significant. Experiments are representative of the results from at least two biological replicates. (B) The pH of apical washes from 27 HAE cultures was tested using an unbuffered 0.9% saline (adjusted to pH 7.4) wash. The medians and interquartile ranges are shown.

When pHAE cells were infected at low multiplicity to perform multicycle growth analysis, the viruses with higher activation pH, the Y7H and A9T mutants, were attenuated, whereas the more acid-stable influenza viral mutants, E21K and WT, replicated to higher titers (Fig. 3A). The area under the curve for the WT was significantly greater than for the A9T (*P* = 0.0295) and Y7H (*P* = 0.0286) mutants.

We hypothesized that the low pH of HA activation (increased stability) could be advantageous to virus infecting pHAE cells by enabling virus to withstand conditions in the extracellular microenvironment, such as pH, mucus, and concentrations of salts and ions, which would not be present in a typical cell culture system. The mammalian upper respiratory tract (URT) is reported to be mildly acidic (28). We tested the pH of apical washes from 27 wells of pHAE cultures derived from two different human donors and measured a median pH of 6.5 (range, 5.8 to 7.0) (Fig. 3B). Viruses with less-pH-stable HA proteins may be more likely to be triggered to undergo premature HA activation in the extracellular environment of pHAEs, rendering them noninfectious at the point of entry into the cell.

To confirm the importance of extracellular microenvironment on viral growth properties in the ALI pHAE cultures, we infected pHAE cells at a low multiplicity in the presence of a liquid culture medium overlay buffered to pH 7.4, aiming to maintain the extracellular apical space under conditions akin to experiments on immortalized cells. Under these conditions, we found that the replication advantage conferred by a low HA pH of activation was abrogated (Fig. 3C). Similarly, in Madin-Darby canine kidney (MDCK) cells, there was no significant difference in viral replication under multicycle conditions (Fig. 3D), even when the extracellular medium was at pH 6.3 (data not shown).

### Virus with an HA activation pH of 5.5 displays increased pathogenicity in mice.

**FIG 4:**
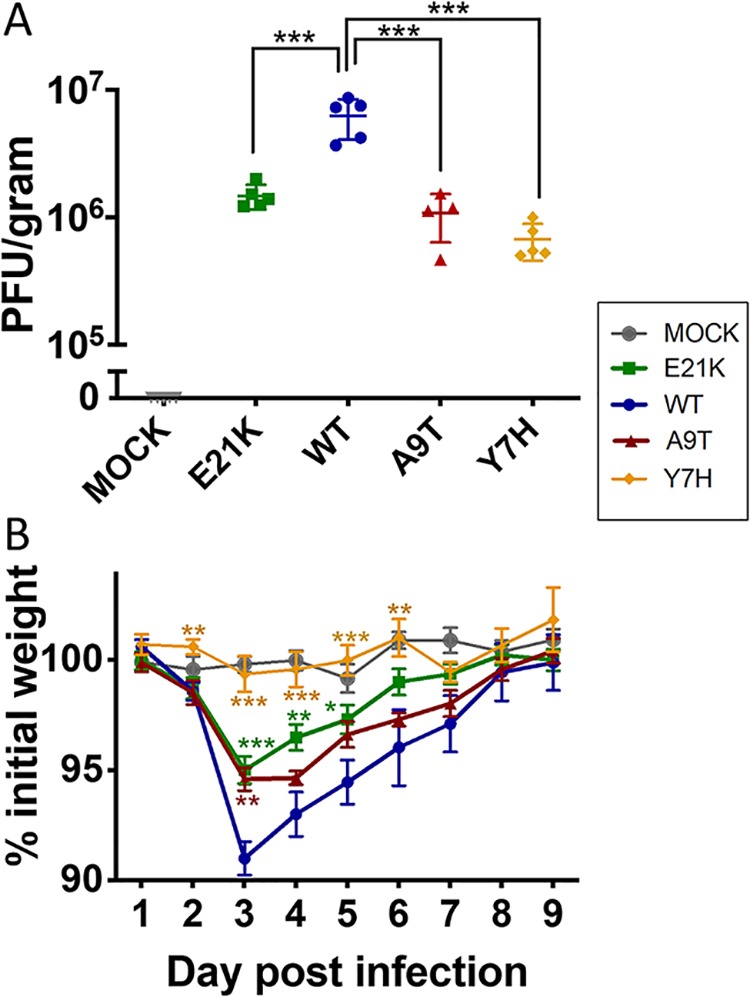
Pathogenicity in mice. Groups of 15 BALB/c mice were inoculated with 200,000 PFU of virus in 40 μl or vehicle (PBS). (A) Mean ± standard deviation (SD) lung viral titers on day 2 postinfection (*n* = 5) were titrated by plaque assay on MDCK cells. (B) Mean (± standard error of the mean [SEM]) percent weight change. One-way ANOVA with Tukey’s posttest was used to compare WT virus to the other viruses. *, *P* < 0.05; **, *P* < 0.01; ***, *P* < 0.001.

To investigate how the observed *in vitro* fitness hierarchies would translate *in vivo*, we infected groups of 15 BALB/c mice with 200,000 PFU of each HA mutant virus. The WT virus replicated to the highest titers in the mouse lung. On day 2, lung viral titers were significantly increased for WT virus compared to those with the E21K (*P* = 0.0069), A9T (*P* = 0.008), and Y7H (*P* = 0.0035) mutants (Fig. 4A), a hierarchy similar to that observed in pHAE cells (WT > E21K > A9T > Y7H) (Fig. 3A). Furthermore, infection with WT virus (pH of fusion 5.5) caused greater weight loss, peaking on day 3 (Fig. 4B), than did the viruses with a fusion pH of 5.3 (E21K mutant, *P* = 0.0004), 5.8 (A9T mutant, *P* = 0.0016), or 5.9 (Y7H mutant, *P* < 0.0001). The area under the weight loss curve for the WT virus was significantly different from those for the E21K (*P* = 0.047) and Y7H (*P* < 0.0001) mutants but failed to reach significance for the A9T mutant (*P* = 0.46).

### Sensitivity to antiviral drugs that act on the HA fusion machinery correlates with HA activation pH.

The stalk region of influenza virus HA is the target for a number of novel therapeutics (17). Examples include directly acting proteins (29, 30), peptides (31, 32), small molecules (20, 33–35), and broadly neutralizing antibodies (BNAbs) (16). A further class of host-targeting drugs, the vacuolar ATPase inhibitors, act on the proton pumps that regulate the pH of host cell endosomes and can inhibit virus by preventing the endosomal acidification required for triggering the fusion process (36–40). Given that the site of action of these drugs is intricately linked to the HA fusion process, we hypothesized that virus might avoid inhibition by mutating to alter its pH of fusion. Additionally, escape mutations within the HA stalk might impact HA activation pH and affect virus phenotype, as described above.

**FIG 5:**
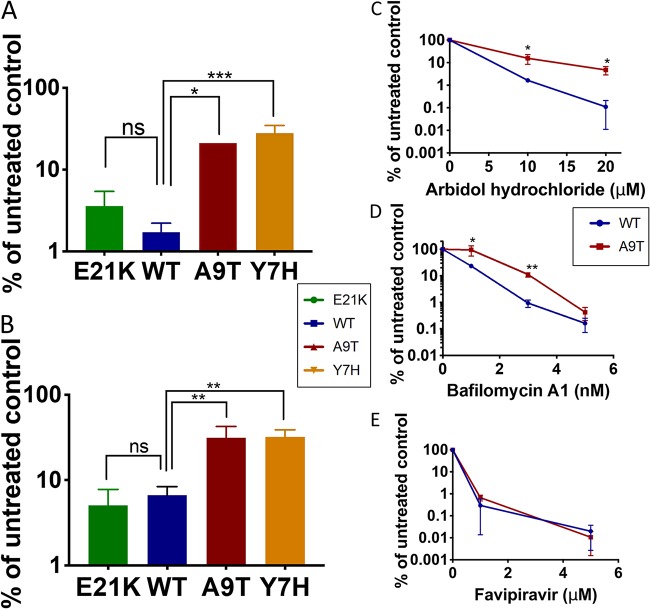
Sensitivity to anti-influenza therapeutics that target the fusogenic ability of hemagglutinin. (A and B) The sensitivity of HA mutants to 20 μM arbidol hydrochloride (a small-molecule fusion inhibitor) (A) and 1 nM bafilomycin (a vacuolar ATPase [vATPase] inhibitor) (B) was assessed by treating virus-infected MDCK cells with drug and titrating virus released at 24 h postinfection by plaque assay on fresh MDCK cells. One-way ANOVA with Tukey’s posttest was used to compare WT virus to the other viruses. *, *P* < 0.05; **, *P* < 0.01; ***, *P* < 0.001; ns, not significant. (C to E) The sensitivity of WT and A9T viruses to arbidol hydrochloride (C), bafilomycin (D), and favipiravir (a nucleoside analogue) (E) at stated doses was tested by treating virus-infected MDCK cells with drug and titrating virus released at 24 h postinfection by plaque assay on fresh MDCK cells. Unpaired Student’s *t* test was used to compare the titers of WT and A9T viruses. *, *P* < 0.05; **, *P* < 0.01. Experiments are representative of at least two biological replicates.

We tested the sensitivity of our panel of HA mutants to arbidol hydrochloride (a small-molecule drug that inhibits fusion [19, 41]) and bafilomycin (a vacuolar ATPase inhibitor that increases endosomal pH [36]). We found that viruses with higher HA activation pH were somewhat less sensitive to these drugs (Fig. 5A and B). To further confirm this finding, we compared the sensitivities of the WT and A9T mutant at multiple doses and found a similar picture, with the A9T mutant displaying around a 2- to 5-fold reduced sensitivity to arbidol hydrochloride (Fig. 5C) and bafilomycin (Fig. 5D). We also tested sensitivity of the WT and A9T mutant to favipiravir, a novel anti-influenza drug with a completely distinct mechanism of action, targeting the viral polymerase. There was no significant difference in the sensitivity of the WT or A9T mutant to favipiravir (Fig. 5E). Our data suggest that antiviral therapeutics that act on the HA stalk/fusion machinery could exert selective pressure on virus leading to changes in HA pH stability.

## DISCUSSION

The emergence of human pandemic H1N1 viruses from a swine reservoir in 2009 provided a unique opportunity to study the evolution of a novel influenza virus following its introduction to the human population. Since 2009, pH1N1 viruses have evolved to become increasingly acid stable, with HA activation pH changing from ∼5.5 in prototypic viruses to ∼5.3 in modern pH1N1 viruses (5, 6, 42). Reports have shown that seasonal human influenza viruses tend to be more acid stable than those that reside in domestic birds and swine (5, 11, 28, 43, 44), implicating that this phenotype has a significant influence on virus fitness in different environments or host species. Here, we assessed the consequences of mutations in pH1N1 HA that alter the pH of activation in a number of different experimental models.

We demonstrate that an acid-labile HA confers a replicative advantage within host cells by enabling efficient viral uncoating in endosomes. A more pH-sensitive virus could uncoat earlier in the endosomal pathway, which might be advantageous through the evasion of innate immune restriction by endosomal interferon-induced transmembrane 2/3 (IFITM2/3) (45). An HA that is too acid stable may not be sufficiently sensitive to trigger pH-dependent uncoating within endosomes and be trafficked for degradation in more acidic lysosomes. Interestingly, the intracellular pH environment that virus is exposed to may vary in different cell types and hosts, meaning that acid stability could restrict a virus’ ability to replicate in certain cell types. For example, Marvin et al. (46) demonstrated that increased pH sensitivity was required for influenza viruses to uncoat and enter the nucleus of macrophages. Others have shown variations in endosomal pH of immortalized cell lines (47, 48) and primary cell cultures (49).

Extracellular pH in different tissues can also impact the successful propagation of viruses that differ in stability. In the absence of a target membrane, HA conformational change results in irreversible virus inactivation and therefore is directly related to virus stability and survivability outside the host cell. In pHAE cells, we found that the attenuation of viruses with pH-unstable HA was abrogated when the extracellular environment was maintained under conditions similar to those of typical cell cultures. Thus, optimum acid stability for the replication of human influenza viruses appears to be determined by a balance between adequate stability to withstand extracellular environmental conditions and adequate pH sensitivity to enable viral uncoating within endosomes once target cells have been successfully reached (50).

**FIG 6:**
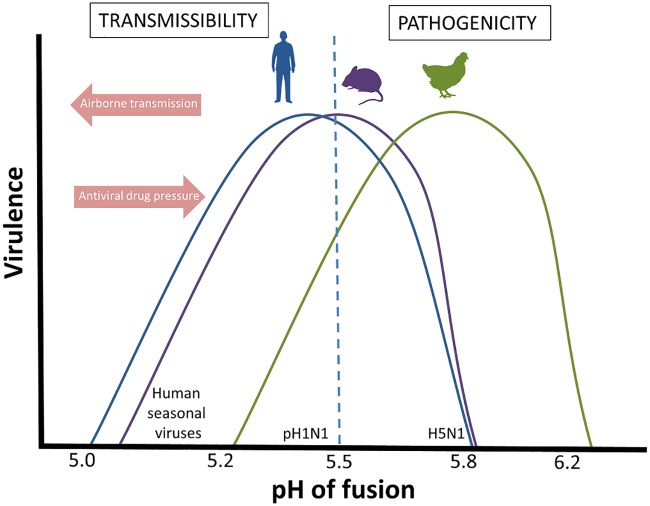
Schematic of the relationship between HA pH of fusion, virulence, and airborne transmissibility. In order to infect and replicate in humans (blue line) or mice (purple line), influenza viruses have an optimum pH of fusion that is lower than that in domestic poultry (green line). This becomes important when assessing for zoonotic influenza viruses with pandemic potential. For an influenza virus to become a successful human pathogen, it must be able to sustain human-to-human airborne transmission, which requires a more stable HA, as is found in human seasonal viruses. Conversely, anti-influenza therapeutics that target the fusogenic ability of the influenza HA may drive virus to evolve an increased pH of fusion, with consequences on the balance between virulence and transmissibility.

We show that pH1N1 influenza virus with a pH of fusion of 5.5 replicated to higher titers in mouse lungs and caused increased weight loss in mice compared to isogenic viruses with either less- or more-stable HAs. While we cannot exclude the possibility that the HA substitutions tested are having pleiotropic effects and that the observed fitness and pathogenicity decreases in mice are not completely attributed to the change in HA pH stability, our observations are consistent with others in the literature and, taken together, suggest an optimum pH for fusion of HA in mice of pH 5.4 to 5.6. For example, using highly pathogenic avian H5N1 viruses, Zaraket et al. (51) demonstrated that virus with a fusion pH of 5.4 displayed greater pathogenicity in mice than those with higher fusion pH (5.6 to 6.3). Russier et al. (5) found that the Y7H pH1N1 mutant with fusion pH of 6.0 was attenuated in mice. Smeenk et al. (52) reported that increased virulence in a mouse-adapted H1N1 strain was mediated in part by HA mutations that lowered the pH stability from 5.8 to 5.6. Conversely, Keleta et al. (53), working with an H3N2 strain, showed increased murine virulence conferred by mutations that increased HA fusion pH from 5.2 to 5.6 following serial passage in mice. In a study by Ilyushina et al. (54), serial passage of a pH1N1 virus in mice did not alter the pH of fusion, which was already at 5.6 in the starting virus. Thus, the optimum pH appears to be determined by the host environment regardless of the viral strain and its baseline HA pH of activation (see Fig. 6 for a schematic representation).

We propose that the evolution of a virus’ optimum acid stability will depend on the nature of its environment, for example, host species, site of replication, and routes of transmission. Emerging avian influenza viruses of human pandemic concern, including the highly pathogenic H5N1 and H7N9 viruses, have evolved less-stable HA proteins (7, 28, 55, 56) (see Fig. 6 for a schematic representation). Possible explanations for this could be differences in the extracellular environment at the site of infection or replication in birds compared to humans. Intracellular differences, for example, endosomal pH and the antiviral activity and distribution of restriction factors, such as IFITMs, might also play a role. Moreover, domestic poultry, which frequently harbors these emerging viruses, tend to be housed in close proximity where direct-contact higher inoculum transmission events are likely to be the predominant mode of viral transmission. The advantage of a pH-stable HA in withstanding environmental stressors is of less importance than for the long-range airborne transmission of human influenza viruses. Evidently, increased understanding of the requirements and implications of HA stability will expand our awareness of how this property may be restricting animal influenza viruses with a higher pH of fusion from crossing the species barrier into humans and aid in the refinement of influenza surveillance and risk assessment for pandemic threats (57, 58).

With the increasing development of therapeutics that directly target the fusogenic ability of HA, understanding the consequences of HA mutations that can both alter HA pH stability and mediate drug resistance is of clinical importance. Interestingly, we show that mutations reducing the pH stability of HA modestly reduce drug sensitivity (∼2- to 5-fold change in inhibitory concentration), which is in line with previous studies (13, 19, 20, 23, 59). While this is unlikely to manifest as clinically relevant resistance, it may indicate that, within a single host, selective drug pressure during treatment could lead to the emergence of variants with reduced HA pH stability. Importantly, the work in this study shows, in the context of pH1N1, that increasing pH of fusion to >5.6 would likely attenuate virus in the human airway, perhaps limiting the clinical consequence of any drug escape mutants. Additionally, it is important to note that drug resistance to stem-targeting drugs could arise via mutations at the drug-binding site and that a change in HA pH stability (either an increase or decrease) could be an unintended consequence of such mutations (23, 60).

Last, a further corollary of our findings is in implications for improving influenza vaccines. Understanding the “optimum” pH of fusion for viral replication in a particular cell type can aid in optimizing vaccine production and effectiveness. Live attenuated influenza vaccines (LAIV) must replicate within the human URT in order to induce a productive immune response, where being more acid stable could enhance viral replication and vaccine immunogenicity (42, 61). On the other hand, the production of cell culture-based influenza vaccines might be optimized by altering viral pH of fusion to maximize *in vitro* replication (48). Mutagenesis of the HA stalk to optimize the HA pH of fusion is an attractive future strategy that could lead to improved take or yield of influenza vaccines. Moreover, our data (Fig. 2B) showing that acid-labile viruses are more efficient at entering the nucleus have relevance for LAIV production. Where a single-cycle replicative assay (such as the focus-forming assay) is used for viral quantification, this could misleadingly overscore the infectious titer of an acid-labile virus and result in unequal quantities of viruses introduced into vaccine preparations. Vaccine manufacturers should be aware of this potential pitfall.

To conclude, our data show that mutations that alter the HA pH of activation have marked effects on virus behavior and point toward an optimum pH of activation for influenza viruses to infect and replicate in humans. This appears to be denoted by a balance between a virus’ need to withstand external environmental stressors while maintaining adequate sensitivity to trigger viral uncoating intracellularly. Understanding the consequences of changes in HA pH stability on viral phenotypes has implications for the rational use of antiviral drugs, improvement of vaccines, and monitoring of pandemic risk.

## MATERIALS AND METHODS

### Cells.

Madin-Darby canine kidney (MDCK) cells and A549 (immortalized human lung epithelial) cells (ATCC) were maintained in Dulbecco’s modified Eagle’s medium (DMEM; Gibco-Life Technologies) supplemented with 10% fetal bovine serum (FBS; Labtech International), 1% penicillin and streptomycin (Gibco-Life Technologies), and 1% nonessential amino acids (Sigma-Aldrich) at 37°C with 5% CO_2_. Primary human airway epithelial cell cultures (MucilAir) were obtained from Epithelix Sàrl and cultured according to the manufacturer’s protocol.

### Viruses.

Wild-type (WT) A/England/195/2009 (pH1N1) virus, typical of an early 2009 pandemic strain, was derived from reverse-genetics systems by *de novo* synthesis (GeneArt) from published sequence data (62). The E21K, A9T, and Y7H point mutations were introduced into a pPol1 plasmid containing the HA of Eng195 using the QuikChange site-directed mutagenesis kit (Stratagene), according to the manufacturer’s protocol. Viruses were generated using a 12-plasmid reverse-genetics system, as previously described (62). All influenza viruses were propagated in flasks of confluent MDCK cells in the presence of DMEM (1% penicillin-streptomycin) and 1 μg/ml tosylsulfonyl phenylalanyl chloromethyl ketone (TPCK)-treated trypsin (Worthington Biosciences) and titrated by a plaque assay on MDCK cells.

### Viral sequencing.

Total RNA was isolated from viral stocks using QIAamp viral RNA minikit (Qiagen) and reverse transcribed using SuperScript III reverse transcriptase (Invitrogen) with random hexamers. The HA gene was amplified using KOD polymerase (Thermo Fisher Scientific), with HA-specific primers (forward [FWD], AGCAAAAGCAGGGGAAAACAAAAGC, and reverse [REV], AGTAGAAACAAGGGTGTTTTTTCTCATGC) and Sanger sequenced.

### Real-time quantitative PCR.

Total RNA was isolated from viral stocks using the QIAamp viral RNA minikit (Qiagen) and reverse transcribed using SuperScript III reverse transcriptase (Invitrogen) with random hexamers. Real-time quantitative PCR (RT-qPCR) for the viral M gene was performed with TaqMan probe 5′-FAM-TYACGCTCACCGTGCCCAGTG-MGBNFQ-3′ (FAM, 6-carboxyfluorescein) (Life Technologies). The primer sequences were AAGACAAGACCAATYCTGTCACCTCT (FWD) and TCTACGYTGCAGTCCYCGCT (REV). Data were analyzed on the Applied Biosystems ViiA real-time PCR system.

### Syncytial formation assay.

MDCK cells at 60% confluence were inoculated with viruses at a multiplicity of infection (MOI) of 10 PFU/cell for 1 h at 37°C and 5% CO_2_ in duplicate. The inoculum was removed, and cells were washed three times with phosphate-buffered saline (PBS) and incubated in DMEM plus 2% fetal calf serum (FCS) for 16 h at 37°C. Cells were treated with 10 μg/ml TPCK-treated trypsin for 15 min at 37°C and then exposed to pH-adjusted morpholineethanesulfonic acid (MES) buffers (100 mM MES, 150 mM NaCl, 0.9mM CaCl_2_, 0.5mM MgCl_2_) for 5 min at 37°C. Buffers were replaced with DMEM plus 10% FCS for 3 h at 37°C, fixed with methanol-acetone (1:1), and stained with Giemsa stain. Visual inspection for syncytia was performed under light microscopy.

### Acid inactivation assay.

Each virus (10^7^ PFU) was mixed with pH-adjusted MES buffer (1:10 dilution) in triplicate and incubated for 15 min at 37°C. The buffer was inactivated with a 10-fold dilution in DMEM and the samples titrated by a plaque assay on MDCK cells.

### Thermostability assay.

Sixty-four hemagglutinating units of each virus was incubated for 30 min at 54°C in triplicate. The HA titer remaining after incubation was tested by a hemagglutination assay.

### *In vitro* viral growth kinetics.

MDCK or A549 cells at 70% confluence were inoculated in triplicate with each virus at an MOI of 0.0001 PFU/cell for multicycle experiments and an MOI of 3 PFU/cell for single-cycle experiments and then incubated for 1 h at 37°C and 5% CO_2_. The inoculum was removed, cells were washed with phosphate-buffered saline (PBS) three times, and 1 ml of serum-free DMEM with 1 μg/ml of TPCK-treated trypsin was added before incubating cells for the indicated time frame. At each time point, 300 μl of medium was removed and replaced with 300 μl DMEM plus 1 μg/ml TPCK-treated trypsin.

For growth curves in pHAE cells, the apical surface of cells was first washed with 200 μl DMEM to remove excess mucus. Triplicate wells of cells were inoculated with each virus at an MOI of 0.001 PFU/cell for 1 h at 37°C and 5% CO_2_. The apical surface was then washed twice with serum-free medium before reincubating at 37°C. At the indicated time points, 200 μl of DMEM was added to the apical surface, incubated for 30 min, removed, and stored at −80°C before being titrated by a plaque assay. For experiments with a DMEM overlay, 200 μl of DMEM was left on the apical surface between time points.

### Virus driven replicon assay.

A549 or 293T cells were transfected with pHSP1-Firefly, a plasmid that directs the production of a virus-like minigenome encoding Firefly luciferase and with three mutations in the 3′-end promoter region that allow amplification by polymerase from infecting virus and *Renilla* luciferase expression plasmid. Sixteen hours later, cells were infected with viruses (E21K mutant, WT, A9T mutant, and Y7H mutant) at an MOI of 1 PFU/cell for 1 h, in triplicate. Inoculum was removed and cells washed gently with PBS before incubating for the indicated time at 37°C and 5% CO_2_. The levels of firefly luciferase (indicating viral replication in the nucleus) and *Renilla* luciferase (a marker of cellular polymerase) were quantified using a luminometer.

### Acid bypass assay.

A549 cells were transfected with pHSP1-Firefly and *Renilla* luciferase expression plasmids. Sixteen hours later, influenza viruses (E21K mutant, WT, A9T mutant, or Y7H mutant) at an MOI of 1 PFU/cell were bound to A549 cells for 1 h at 4°C, in triplicate. Cells were washed twice with chilled (4°C) PBS to remove any unbound virus particles and then incubated with prewarmed MES buffer with pH adjusted to 5.0 at 37°C for 2 min to induce virus fusion at the cell surface. Cells were washed twice with chilled PBS and then incubated for 24 h at 37°C with DMEM plus 20 mM NH_4_Cl and 50 mM HEPES to block viral entry via the endocytic pathway. The levels of firefly and *Renilla* luciferase were quantified using a luminometer. A negative (no-fusion) control treating with pH 7.4 buffer followed by DMEM plus NH_4_Cl and HEPES and a positive (endosomal fusion) control treating with pH 7.4 buffer followed by DMEM plus 50mM HEPES were carried out simultaneously.

### Mouse experiments.

Fifteen female BALB/c mice (6 to 8 weeks old) were anesthetized with nebulized isoflurane and intranasally inoculated with 2 × 10^5^ PFU of each virus in 40 μl of PBS or a mock control. Mice were weighed daily and sacrificed if >20% weight loss occurred. At day 2 postinfection, 5 mice from each group were sacrificed and the lungs harvested and weighed. The lung homogenate was titrated by plaque assay on MDCK cells.

### Drug sensitivity.

MDCK cells at 70% confluence were infected with each viral mutant at an MOI of 0.01 PFU/cell in triplicate and incubated for 1 h at 37°C and 5% CO_2_. The inoculum was removed, cells were washed with PBS three times, and 1 ml of serum-free DMEM with 1 μg/ml TPCK-treated trypsin, with or without drug at the stated dose, was added before incubating the cells for 24 h. The drugs tested included the vacuolar ATPase inhibitor bafilomycin (Sigma-Aldrich), the HA stalk-targeting fusion inhibitor arbidol hydrochloride (19, 41) (Carbosynth Ltd.), and the nucleoside analogue favipiravir (Toyama Chemical) (63). The remaining infectivity was titrated by a plaque assay on MDCK cells.

### Statistical analysis.

All data analysis and graphs were prepared using GraphPad Prism (GraphPad Software, San Diego, CA). Data are presented as the mean ± standard deviation (SD) of the results from three or more experiments, unless otherwise stated. One-way analysis of variance (ANOVA) with a posttest for multiple comparisons was performed to compare the WT to the mutant viruses. For comparisons between two groups (WT versus A9T mutant), unpaired Student’s *t* test was used. The area under the curve, where performed, was compared by one-way ANOVA. A *P* value of <0.05 was considered significant.

### Ethics statement.

All work was approved by the local genetic manipulation (GM) safety committee of Imperial College London, St. Mary’s Campus (center number GM77) and the Health and Safety Executive of the United Kingdom. Animal work was performed under a United Kingdom Home Office License, PPL 70/7501, in accordance with the approved guidelines, under the Animals (Scientific Procedures) Act 1986 (ASPA).
